# The Traditional Chinese Medicine Formula *Modified Wu Bao San* Induces Cell Cycle Arrest and Apoptosis through Restoration of Retinoblastoma Protein Function and Inhibition of Aurora Kinase Activity

**DOI:** 10.7150/jca.114495

**Published:** 2025-07-11

**Authors:** Chih-Jui Chang, Cheng-Chia Wu, Yi-Jen Hsieh, Jhong-Kuei Chen, Yen-Lun Kung, Wan-Ting Tsai, Hsuan-Shun Huang, Shinn-Zong Lin, Tsung-Jung Ho

**Affiliations:** 1 Integration Center of Traditional Chinese and Modern Medicine, Hualien Tzu Chi Hospital, Hualien, Taiwan.; 2 Department of Chinese Medicine, Hualien Tzu Chi Hospital, Hualien, Taiwan.; 3 School of Post-Baccalaureate Chinese Medicine, Tzu Chi University, Hualien, Taiwan.; 4 Department of Molecular Biology and Human Genetics, Tzu Chi University, Hualien, Taiwan.; 5 Bioinnovation Center, Buddhist Tzu Chi Medical Foundation, Hualien, Taiwan.; 6 Department of Neurosurgery, Hualien Tzu Chi Hospital, Buddhist Tzu Chi Medical Foundation, Hualien, Taiwan.; 7 Center for Prevention and Therapy of Gynecological Cancers, Department of Research, Hualien Tzu Chi Hospital, Buddhist Tzu Chi Medical Foundation, Hualien 970, Taiwan.; 8 Institute of Medical Sciences, Tzu Chi University, Hualien, Taiwan.

**Keywords:** traditional Chinese medicine, Wu Bao San, Chia Wei Wu Pao San, cancer, Aurora kinases, retinoblastoma, cell cycle, apoptosis

## Abstract

**Background:** Traditional Chinese Medicine (TCM) has a long-standing history in treating various diseases, including cancer. Wu Bao San is a traditional formula known for its ability to clear heat, detoxify, relieve cough and phlegm and treat tumours. The modified Wu Bao San (MWBS) is a conventional medicine prescribed by doctors of Chinese medicine. However, the molecular mechanisms underlying the anticancer effects remain unclear owing to the complexity of the components of traditional Chinese medicine, making it challenging to pinpoint specific molecular targets or pathways underlying their clinical effect.

**Methods:** In this study, we used an MTT assay, flow cytometry, indirect immunofluoresence imaging, and western blotting to analyse the viability, cell cycle profiles, morphology, and protein levels of cells after MWBSE treatment.

**Results:** Our results showed that MWBSE has multiple anticancer effects. First, MWBSE induces cytokinesis failure by inhibiting Aurora kinase, leading cells to commit to cell death. Additionally, MWBSE reduces the phosphorylation of Rb, restoring its function and resulting in cell cycle arrest. The overexpression of Aurora kinases plays a pivotal role in sustaining malignant cell behaviors such as uncontrolled growth, colony formation, and tumor development. The retinoblastoma protein (Rb) is a key tumor suppressor that regulates the cell cycle, particularly the transition from the G1 to the S phase due to its significant involvement in tumor biology. Aurora kinases and Rb have emerged as promising targets for cancer therapy.

**Conclusion:** Our findings provide a mechanistic explanation of the targets and anti-tumor pathways of MWBSE.

## Introduction

The understanding of tumors in traditional Chinese medicine (TCM) dates back to the Yin-Shang period of ancient China. TCM practitioners have long attributed cancer development to imbalances involving "deficiency, toxins, phlegm, and blood stasis," factors believed to directly or indirectly contribute to tumor formation. The well-known traditional formula Wu Bao San was originally documented in the Medical Compendium of the Golden Mirror (Yi Zong Jin Jian). This formula is traditionally composed of cinnabar, amber, pearl, borneol, and stalactite and is prescribed for its activity of heat-clearing, calm the mind, detoxicating, relieving cough and phlegm, promoting urination, improving vision. However, owing to the toxicity of cinnabar, careful preparation is needed to reduce their harmful effects 1.

To address this, pharmaceutical companies modified the Wu Bao San formula by replacing cinnabar with porcine gallbladder and Calculus bovis to provide similar therapeutic effects without the associated toxicity risks. Today, modified Wu Bao San primarily includes amber, pearl, borneol, starch, stalactite, porcine gallbladder, and Calculus bovis, offering a safer alternative while retaining the formula's healing properties.

The Modified Wu Bao San formula is composed of several traditional Chinese medicinal ingredients, each with unique properties that contribute to its therapeutic effects. Amber, which is sweet and neutral in nature, is used for relieving convulsions, and promoting blood circulation to alleviate blood stasis. It is often used for sedation, diuresis, and blood flow activation 2-5. Pearl, which is cold and sweet in nature, has detoxifying effects. It clears heat, alleviates inflammation and pain. Pearl is commonly used to treat symptoms of sore throat, swollen eyes. Borneol is cool and spicy in nature. It reduces swelling and pain 5, 6. Stalactite has detoxifying effects. It is effective for reducing symptoms such as mouth sores, sore throat, swollen gums, and red, swollen eyes. Pig bile is valued for its ability to clearing heat and toxins and is known for its ability to brighten the eyes. Bezoar (Calculus bovis) helps clearing heat, detoxify, resolve phlegm, and open the orifices 7. On the basis of these properties and supporting clinical research, the modified Wu Bao San formula is used to heat-clearing and detoxicating, treating ulcers, promoting blood circulation, resolving blood stasis.

The Aurora kinases, a family of serine/threonine kinases, consists of three members: Aurora A (AURKA), Aurora B (AURKB), and Aurora C (AURKC). These kinases are key regulators of essential cellular processes from the onset of mitosis to the final step of cytokinesis. Despite their high homology, Aurora A and Aurora B exhibit significant differences in terms of their localization and function. Aurora A is associated primarily with spindle poles and plays a critical role in regulating entry into mitosis, maturation and separation of centrosomes, and spindle assembly. Aurora B, on the other hand, is part of the chromosomal passenger complex (CPC), which also includes the scaffold protein INCENP and the localization subunits Survivin and Borealin/Dasra B. The CPC localizes to centromeres before metaphase and then relocates to the spindle midzone, the equatorial cortex, and the midbody during anaphase and cytokinesis. Aurora B's functions include regulating chromosome-microtubule interactions, sister chromatid cohesion, spindle stability, and cytokinesis. Importantly, Aurora B is highly expressed in various cancers and is associated with poor prognosis 8-10. Inhibition of Aurora B can induce apoptosis and significantly reduce the survival of cancer cells in both *in vitro* and *in vivo* studies 11-13. Aurora C, which closely resembles Aurora B, is less studied and is expressed primarily in the testes, where its function remains under investigation. Several other proteins, such as Polo-like kinases (Plk1) and the RhoA GTPase signalling pathway, are also crucial for the successful completion of cytokinesis 14, 15. Inhibiting these key proteins can directly block cytokinesis, causing cell division to fail. This mechanism has been recognized as a potential therapeutic target in cancer treatments, where disrupting cytokinesis in cancer cells can inhibit tumor growth and proliferation.

The retinoblastoma protein (Rb) plays a pivotal role in regulating the cell cycle, particularly at the G1 to S phase transition, ensuring that cells only proceed to DNA synthesis when conditions are favour 16. Initially discovered due to its mutation in retinoblastoma, a rare childhood eye cancer, Rb has since been implicated in a variety of cancers due to its central role as a tumor suppressor. Mutations or dysregulation of Rb disrupts its normal function, contributing to uncontrolled cell proliferation and, ultimately, oncogenesis 17.

In this study, we demonstrated that the ethanol extract of Wu Bao San (MWBSE) has multiple anticancer effects. MWBSE restores Rb function, inhibits Aurora kinase activity, induces cytokinesis failure, and promotes apoptosis in cancer cells. Our findings elucidate the molecular mechanisms of Wu Bao San, highlighting its potential for anti-cancer applications.

## Materials and Methods

### Formula extraction

The Chinese medicine formula 'Modified Wu Bao San' was obtained from Chuan Feng Tang Pharmaceutical Company, Taiwan (manufacturing batch number: R2012101; validity period: 2025/08/10). The formula was processed into powder form and then extracted with ethyl alcohol through refluxing. Three grams of MWBS powder were homogenized in a 250 mL round-bottom flask containing 20 mL of ethyl alcohol and placed in a water bath at a constant temperature of 50°C for 8 hours. After extraction, the solution was filtered and concentrated using a rotary evaporator. The crude extract was then lyophilized in a freeze-drier and stored at -20°C until use.

### Cell lines and culture

Human breast adenocarcinoma (grade III) (MDA-MB-231), human cervical epithelioid carcinoma (HeLa), and human hepatocellular carcinoma (HuH7) cell lines were purchased from the Food Industry Research and Development Institute (Hsinchu, Taiwan). The cells were grown according to the supplier's instructions in media supplemented with 10% fetal bovine serum (FBS) and maintained at 37°C in a humidified atmosphere with 5% CO₂.

### Analysis of cell proliferation

The percentage of growth inhibition was determined via the MTT cell proliferation/viability assay. A total of 1000-2000 cells/well were seeded in a 96-well plate and incubated for approximately 16 hours to allow for cell attachment. The cells were then treated with various concentrations of MWBSE and incubated for an additional 3 days at 37°C. Each concentration group was tested in triplicate. After treatment, the test solutions were replaced with culture medium containing 500 μg/mL MTT (Thiazolyl Blue Formazan, Sigma, Taiwan) for an additional 4-6 hours. The supernatant was removed, and 200 μL of DMSO was added to each well to dissolve the formazan crystals. Optical density (OD) was measured via an ELISA reader (SUNRISE, TECAN, Switzerland) at a wavelength of 570 nm. The mean and standard deviation for each group were calculated, and the OD readings were adjusted by subtracting the blank control (background). Relative survival rate was calculated as follows: The relative survival rate = [OD (treatment group) / OD (negative control)] × 100.

### Western blotting

Reagents were purchased from Sigma unless otherwise stated. The antibodies used for immunoblot were as follow: anti-alpha tubulin (mouse mAb B512; Sigma, Taiwan); anti-Aurora A polyclonal antibody (Cell signaling, Taiwan); anti-phoso Aurora A, B, C polyclonal antibody (Cell signaling, Taiwan). For each of three independent experiments, total protein from HeLa cells was extracted following treatment with RIPA lysis buffer supplemented with a complete protease and phosphatase inhibitor cocktail. After centrifugation at 13,000 rpm for 30 minutes, the protein content was measured via the Bio-Rad protein assay reagent (Bio-Rad Laboratories, Hertfordshire, UK), with BSA used as the standard. Equal amounts of denatured proteins were separated by SDS-PAGE and transferred onto PVDF membranes, which were blocked with 5% nonfat milk. The membranes were incubated overnight at 4°C with primary antibodies, followed by incubation with horseradish peroxidase (HRP)-conjugated secondary antibodies. Detection was performed via enhanced chemiluminescence (ECL) (GE), and chemiluminescent signals were visualized with a UVP BioSpectrum 810 Imaging System (Thermo Fisher Scientific). Band quantification was carried out via ImageJ software.

### FACS analysis

The cells treated with various concentrations of MWBSE or DMSO were harvested at 24, 48, and 72 hours. Both floating and adherent cells were collected and fixed in cold 70% ethanol at 4°C overnight. After being washed, the cells were treated with RNase and stained with propidium iodide (PI) for 1 hour in the dark at room temperature. Flow cytometric analysis was conducted via a FACScalibur flow cytometer (Becton Dickinson), and cell cycle distribution was analysed via Cell Quest software (Becton Dickinson). Each experiment was conducted in triplicate.

### Immunofluorescence

For immunostaining, the cells were fixed with 4% paraformaldehyde in PBS for 10 minutes at room temperature, permeabilized with 0.1% Triton X-100 in PBS for 5 minutes, and rinsed with PBS. Blocking was performed in PBS containing 10% FBS for 30 minutes at room temperature. Primary antibody incubations were conducted in PBS for 1 hour, followed by four 10-minute washes with PBS at room temperature. DNA was stained with 0.1 mg/mL DAPI for 5 minutes at room temperature and rinsed with PBS. The slides were mounted with Vectashield mounting medium (Vectra) and sealed with nail varnish. Images were processed via ImageJ software.

### Statistical analysis

All experiments were performed at least in triplicate. The data are presented as the means ± standard deviations (SD), and p-values were analysed using Student's t-test, with analysis of variance (ANOVA) applied where appropriate.

## Results

### The ethanol extract of MWBS (MWBSE) effectively suppresses the proliferation of several types of human cancer cells in a dose- and time-dependent manner

**Figure 1:**
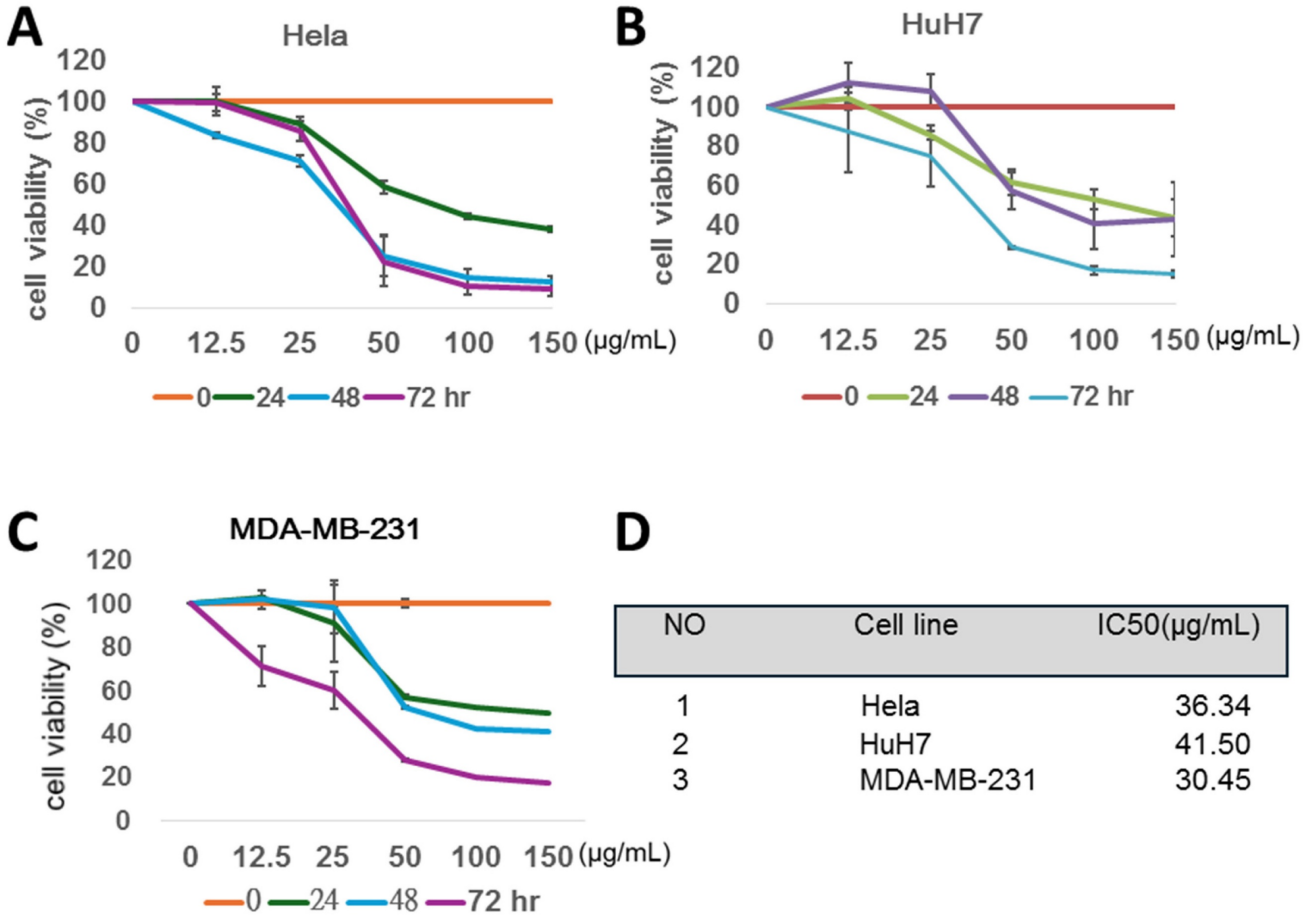
** MWBSE inhibits cell viability of three cancer cell lines. (A-C)** The human carcinoma cell lines HeLa, HuH7, and MDA-MB-231 were treated with various concentrations of MWBSE (0, 12.5, 25, 50, 100, or 150 μg/mL). DMSO was used as a control. The cells were harvested at different time points for MTT assay. **(D)** The IC₅₀ values for MWBSE in the three cancer cell lines after 72 hours of treatment. The results were based on at least three independent experiments.

The anti-proliferative activity of MWBSE was investigated in three different human cancer cell lines—HeLa, HuH7, and MDA-MB-231 (Fig. 1). MTT assays were used to analyse cell viability after MWBSE treatment. The cells were treated with various concentrations of MWBSE (0, 12.5, 25, 50, 100, or 150 μg/mL) for 24, 48, and 72 hours. This cytotoxic effect was observed in a dose- and time-dependent manner (Figure 1A-C). Our results showed that MWBSE exhibited significant cytotoxic activity across all three cancer cell lines, with IC50 values of 36.34 μg/mL for HeLa cells, 41.50 μg/mL for HuH7 cells, and 30.45 μg/mL for MDA-MB-231 (Figure 1D). MWBSE can effectively inhibit the growth of several types of human cancer cells.

### MWBSE induces cell cycle arrest and causes polyploidy in human cervical epithelioid carcinoma cells

**Figure 2:**
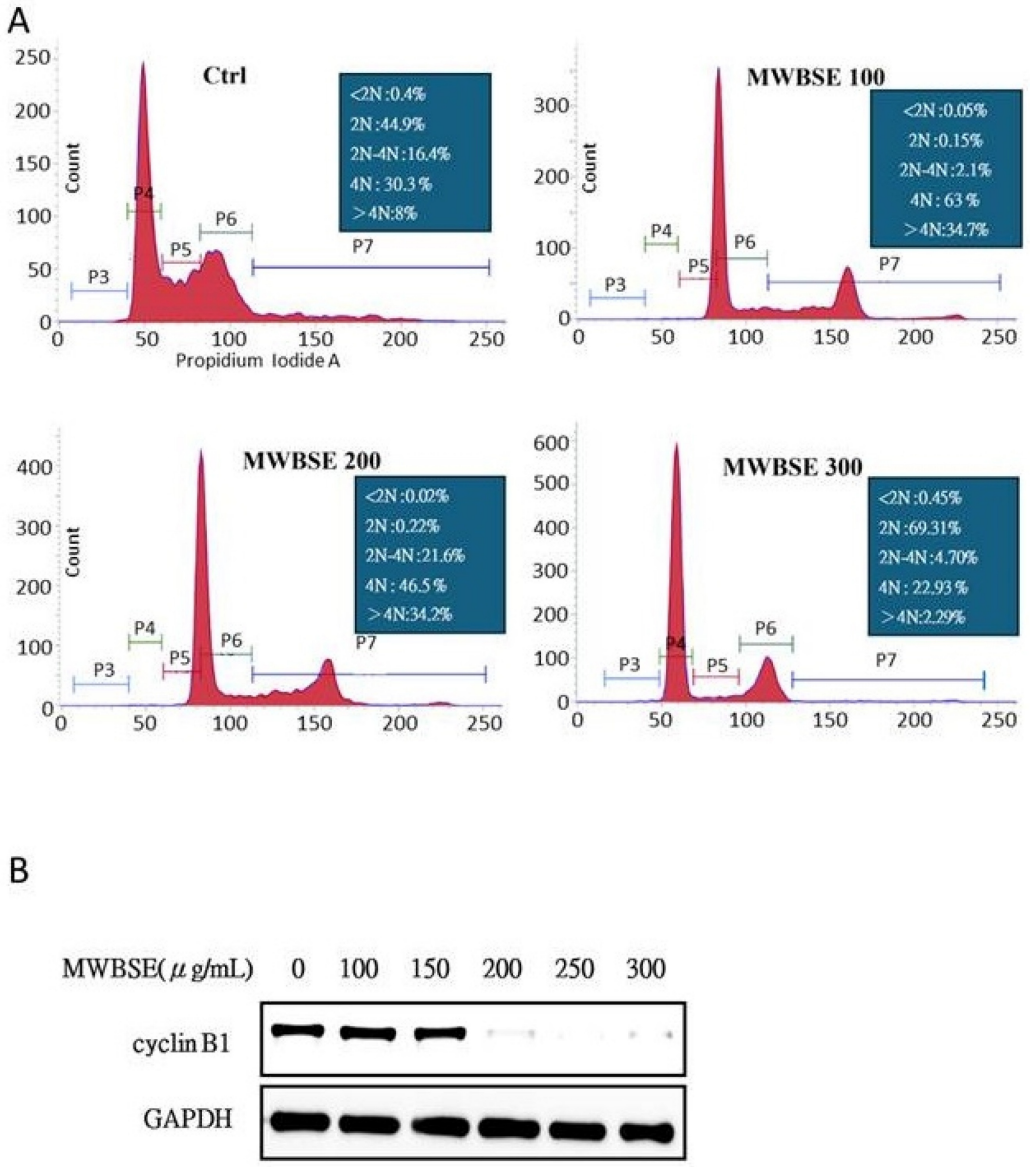
** MWBSE induces cell cycle arrest in HeLa cells. (A)** Flow cytometry analysis of the cell cycle distribution of HeLa cells treated with 100-300 μg/mL MWBSE for 24 hours, revealing shifts in cell cycle phases. **(B)** Western blot analysis showing a reduction in Cyclin B1 levels in MWBSE-treated HeLa cells, with GAPDH as the loading control. The western blot data represents averages from three independent experiments. The flow cytometry data represents averages from two independent experiments.

To investigate the mechanism by which MWBSE inhibits cell proliferation, flow cytometry was used to analyse the cell cycle distribution of HeLa cells. The population of G2/M or diploid/polyploid cells increased significantly when the cells were exposed to a low concentration (100 μg/mL) of MWBSE (Fig. 2). Further analysis revealed that MWBSE treatment led to a reduction in the expression levels of cyclin B1. These findings suggest that the inhibitory effect of low MWBSE concentrations on cell proliferation is linked to the downregulation of cyclin B1, resulting in cell cycle arrest. The cells treated with higher concentrations (300 μg/mL) of MWBSE exhibited cell cycle arrest in the G1 phase, which was accompanied by a decrease in the number of cells in the S phase. These findings suggest that high concentrations of MWBSE induce distinct cell cycle arrest in the G1 phase, further contributing to the inhibition of cell proliferation. In summary, treatment with low concentrations of MWBSE appears to induce G2/M phase arrest. However, cells treated with a high concentration of MWBSE “slipped” into the G1 phase through the downregulation of cyclin B1, promoting polyploidy (Fig. 2B).

### MWBSE-induced apoptosis in cervical epithelioid carcinoma cells

**Figure 3:**
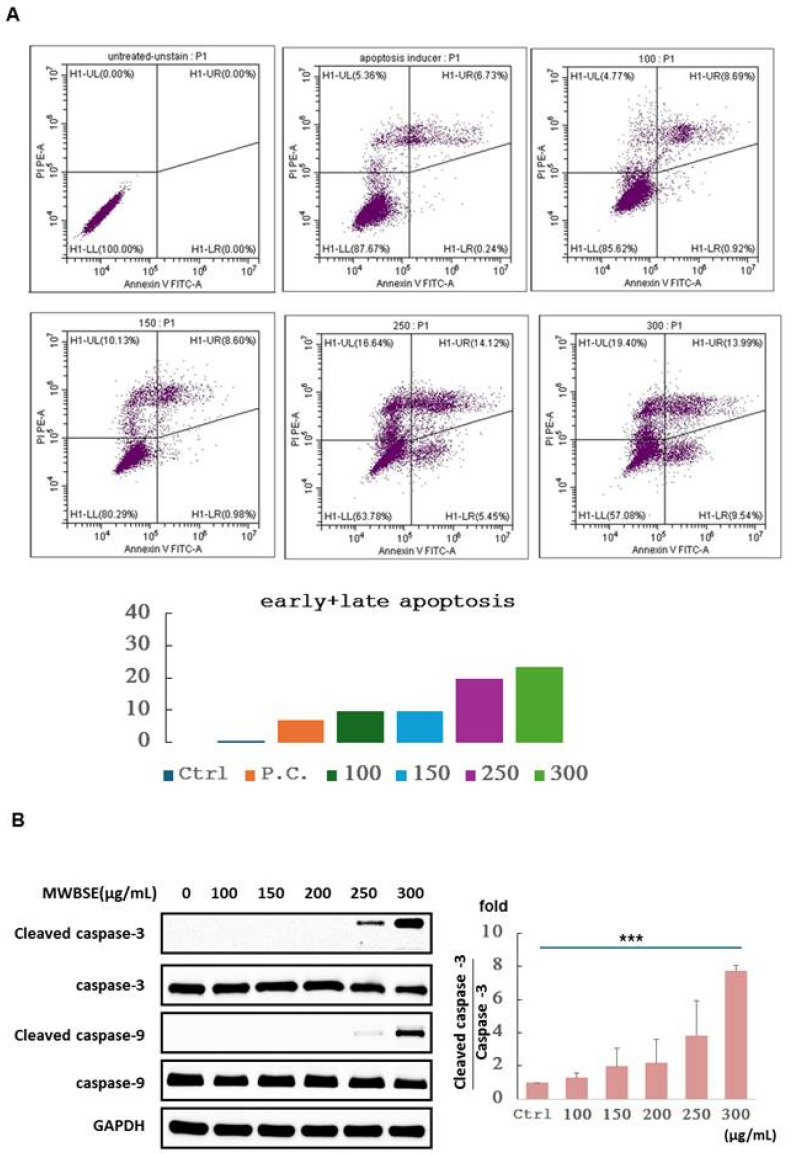
** Induction of apoptosis by MWBSE in HeLa cells. (A)** HeLa cells were treated with various concentrations of MWBSE for 24 hours, and apoptosis was assessed using the Annexin V: AbFluor™ 488/PI double staining assay via flow cytometry. **(B)** Western blot analysis showing increased cleavage of caspase-3, caspase-9, and PARP in response to MWBSE. Treatment with MWBSE resulted in increased cleavage of caspase-3, caspase-9, and PARP, indicating activation of the caspase-dependent apoptotic pathway. GAPDH served as the loading control. The western blot data were from three independent experiments. The data are presented as the means ± SEMs (*p < 0.05, **p < 0.01, ***p < 0.001 compared with controls).

To determine whether MWBSE inhibits the viability of cervical epithelioid carcinoma cells by inducing apoptosis, HeLa cells were treated with various concentrations of MWBSE. Apoptosis induction was assessed via an Annexin V: AbFluor™ 488/PI double-staining assay via flow cytometry after 24 hours of treatment. The results demonstrated a dose-dependent increase in the percentage of apoptotic cells (Figure 3A). Additionally, the analysis revealed an apoptotic response characterized by increases in cleaved caspase-3, and cleaved caspase-9 (Figure 3B). These findings indicate that MWBSE inhibits the growth of cervical epithelioid carcinoma cells through the activation of caspase-dependent apoptosis.

### Cytokinesis failure and multinucleate phenotype induced by MWBSE

Polyploidy, a condition in which a cell has more than two complete sets of chromosomes, can result from failures in early mitosis or cytokinesis. During normal cell division, chromosomes duplicate during the S phase and separate during mitosis. If errors occur during anaphase (e.g., if the mitotic spindle fails to segregate chromosomes properly), the duplicated chromosomes may not separate correctly into two daughter cells. Instead, all chromosomes may remain within a single nucleus, doubling the chromosome set without division and resulting in polyploidy. After mitosis, cells typically complete division through cytokinesis. If cytokinesis fails, the cell does not divide into two separate cells, resulting in one large cell with either two nuclei or a single nucleus with duplicated chromosomes 18.

**Figure 4:**
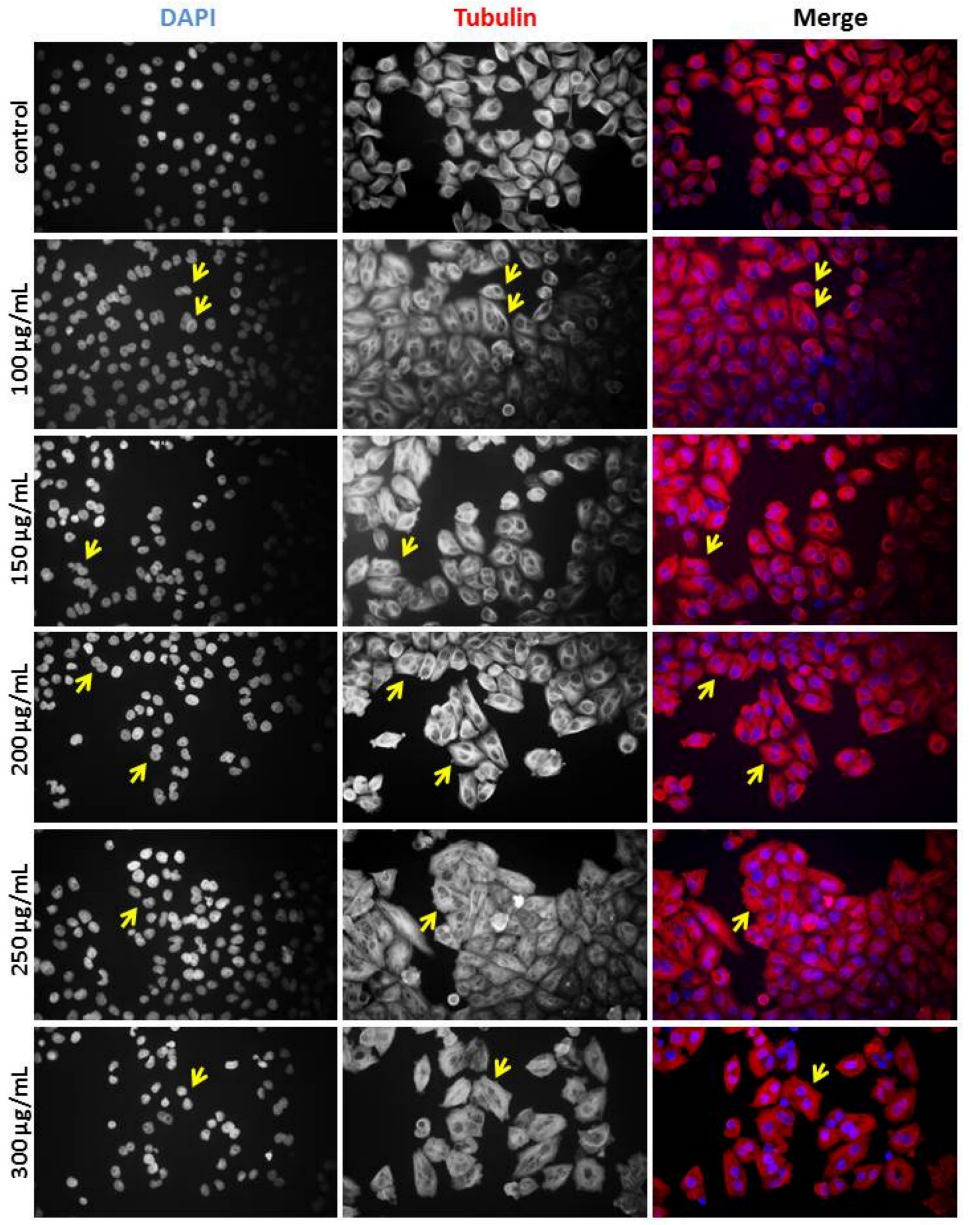
** MWBSE induces cytokinesis failure in HeLa cells.** HeLa cells were treated with MWBSE (100-300 μg/mL) for 24 hours and then subjected to immunostaining. DAPI was used for DNA visualization, and anti-tubulin staining was used to highlight the microtubule structure. Yellow arrows indicate examples of multi-nuclei cells.

To determine whether the diploid/polyploid cells resulted from mitotic failure or cytokinesis failure, we performed immunostaining to analyse cell morphology. Immunofluorescence staining of alpha-tubulin and DAPI-stained DNA was used to examine the structure of microtubules and nuclei. In the control experiments, interphase cells contained a single nucleus. However, the population of binucleated cells increased significantly upon treatment with MWBSE (Fig. 4). These results suggest that MWBSE induces cytokinesis failure.

### MWBSE inhibits aurora kinase activity

**Figure 5:**
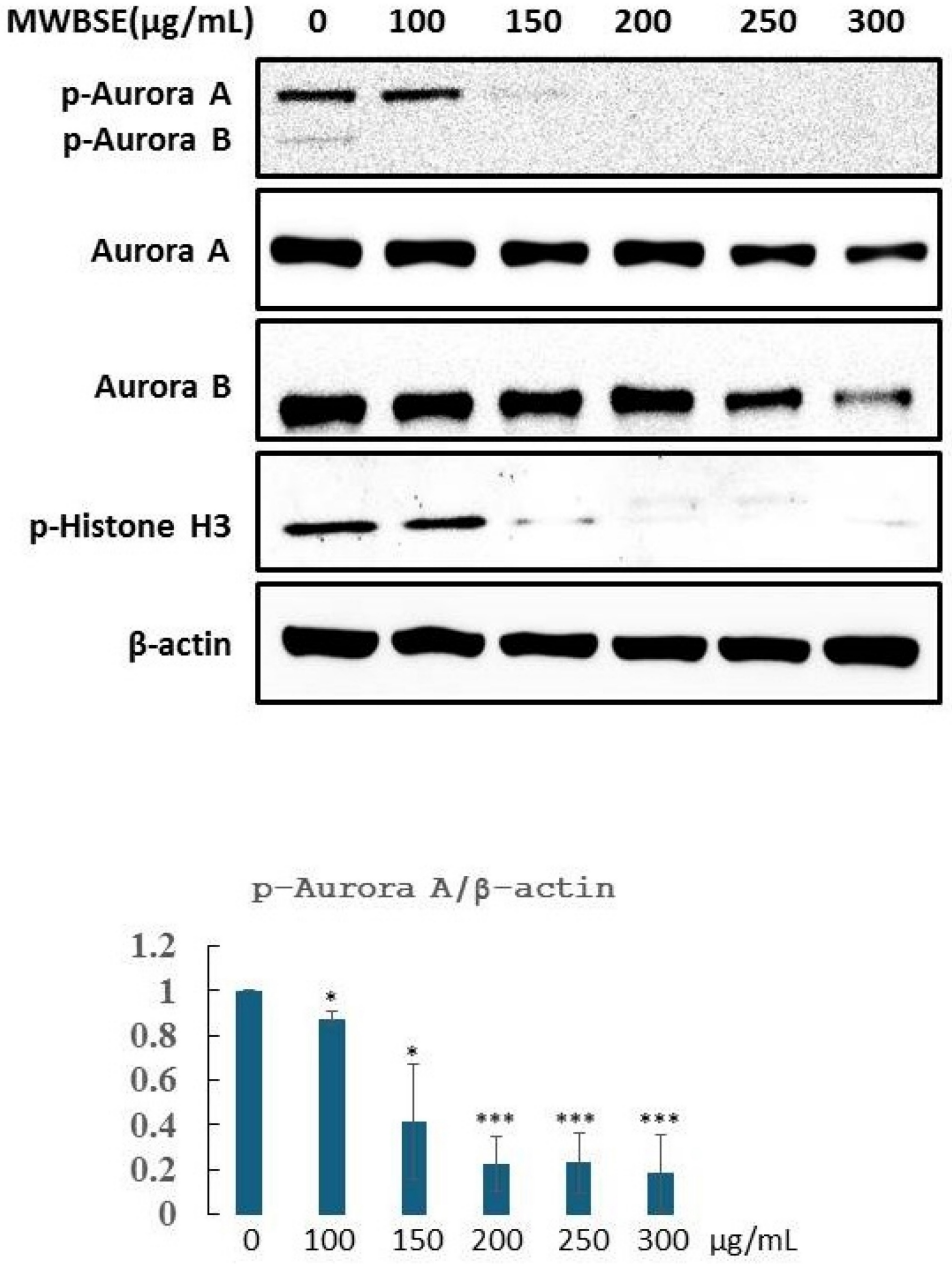
** MWBSE inhibits aurora kinase phosphorylation in HeLa cells.** HeLa cells were arrested in mitosis and treated with MWBSE for 24 hours. Western blot analysis shows total protein and phosphorylated (active) levels of Aurora kinases after treatment. The data were averaged from three independent experiments, with β-actin used as a loading control. The data are presented as the means ± SEMs (*p < 0.05, **p < 0.01, ***p < 0.001 compared with controls).

Aurora B is essential for cytokinesis. Phosphorylation of T232 in the T-loop of the Aurora B kinase domain is critical for its full activation 19. Aurora B specifically phosphorylates serine residues on histone H3, with phosphorylation at serine 10 being most prominent during the early phases of mitosis (prophase) and continuing through metaphase. This phosphorylation event is closely associated with chromatin condensation, which is essential for proper chromosome segregation (Hsu et al., 2000). MWBSE treatment resulted in cytokinesis failure, leading to the formation of diploid cells (Fig. 2, 3). These findings suggest that Aurora kinase may be a target of MWBSE. To confirm the inhibitory effect of MWBSE on Aurora kinase activity in HeLa cells, we analysed the phosphorylation levels and activity of Aurora B following MWBSE treatment. The results showed that MWBSE reduced the phosphorylation of Aurora B kinase at Thr232 and of histone H3 at Ser10 in a dose-dependent manner. Additionally, the protein levels of Aurora kinases were reduced (Fig. 5). These findings indicate that MWBSE inhibits Aurora B activity and induces cytokinesis failure, leading to the formation of diploid/polyploid cells.

### MWBSE restores Rb function by dephosphorylating Rb and further cleaves Bax

**Figure 6:**
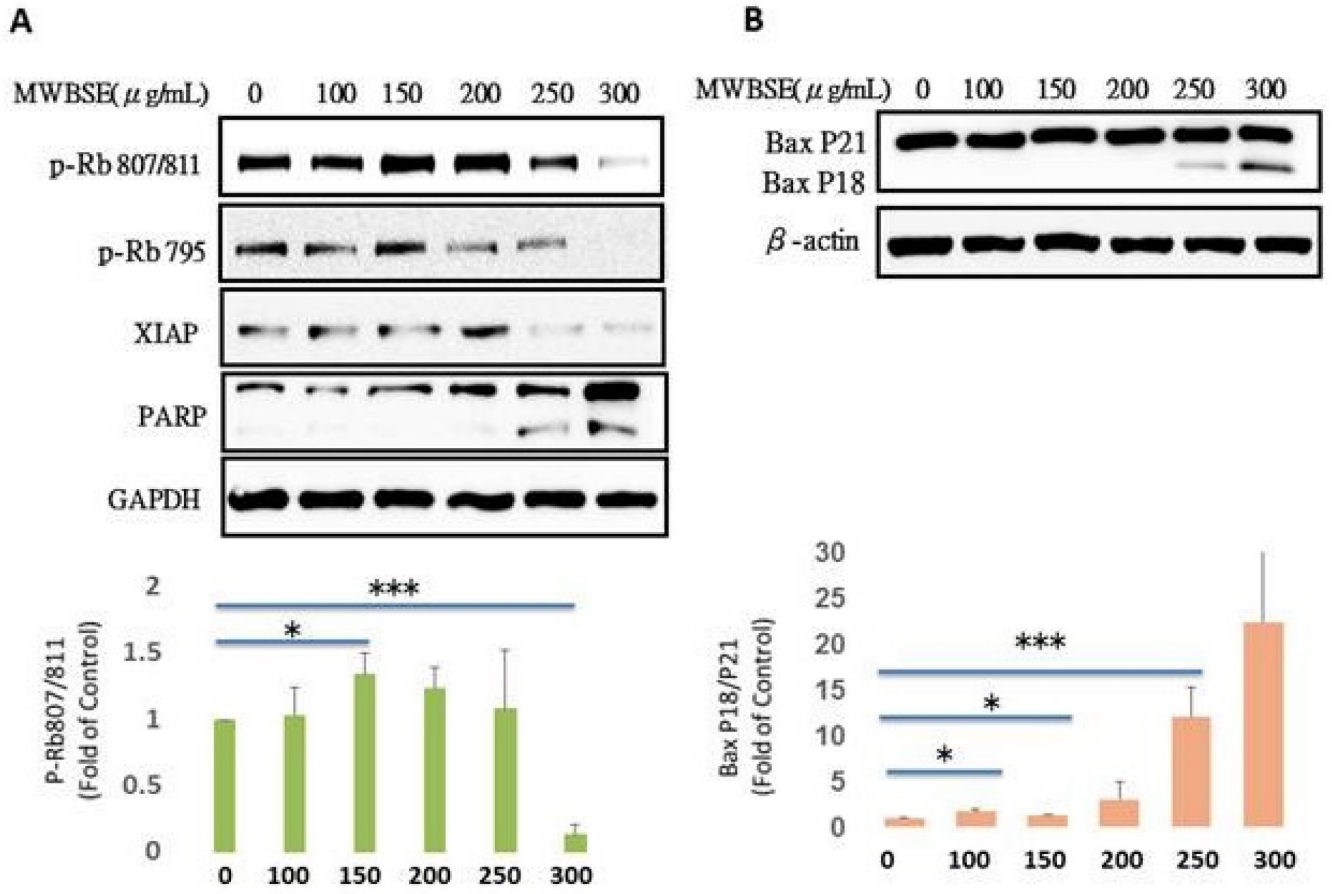
** Reduction in Rb phosphorylation and increase in the Bax P18 protein by MWBSE.** HeLa cells were treated with MWBSE for 24 hours. **(A)** Western blot showing reduced phosphorylation of Rb at the Ser807/811 and Ser795 sites. **(B)** Increased levels of the Bax P18 protein following MWBSE treatment. GAPDH and β-actin were used as loading controls. Data represent averages from three experiments, presented as the means ± SEMs (*p < 0.05, **p < 0.01, ***p < 0.001 compared with controls).

The retinoblastoma protein (Rb) is a key tumor suppressor that regulates the cell cycle, particularly the transition from the G1 phase to the S phase. Rb primarily controls the cell cycle by inhibiting the activity of E2F transcription factors, which are essential for the expression of genes involved in DNA replication and cell cycle progression. In its active, hypophosphorylated state, Rb binds to E2F, preventing the transcription of these genes. However, when cells receive proliferative signals, cyclin-dependent kinases (CDKs) phosphorylate Rb, leading to the release of E2F and promoting cell cycle progression into the S phase (Dick & Rubin, 2013). MWBSE treatment increased the population of diploid cells (Fig. 4) and arrested cells in the G0/G1 phase, accompanied by a reduction in S phase cells (Fig. 2). Since Rb is a key regulator of the cell cycle at the G1 phase, we investigated the possibility that Rb might be affected by MWBSE. To test this hypothesis, the phosphorylation levels of Rb were analysed after MWBSE treatment. MWBSE treatment decreased Rb phosphorylation (Fig. 6A), indicating that MWBSE can reduce the phosphorylation status of Rb and arrest the cell cycle.

Bax (Bcl-2-associated X protein) is a critical regulator of apoptosis within the Bcl-2 protein family. Bax exists in two forms, namely full-length Bax p21 and truncated Bax p18, both of which have been observed in tumor cell lines undergoing apoptosis after treatment with various chemotherapeutic or biological agents. Bax p21 is responsible for initiating mitochondrial permeabilization, whereas Bax p18 enhances and accelerates this process, leading to a swift and irreversible commitment to cell death. We also observed that Bax p18 increased following MWBSE treatment (Fig. 6B).

## Discussion

Traditional Chinese medicine (TCM) has unique advantages in treating cancers because of its comprehensive pharmacological effects through multiple channels, levels, and targets. TCM formulas play essential roles in improving overall health, alleviating symptoms, and supporting modern medical treatments through a diagnostic approach known as "syndrome differentiation and treatment." Many Chinese herbs exhibit direct or indirect antitumor properties. For example, herbs such as *Scutellaria baicalensis*, *Hedyotis diffusa*, and *Scutellaria barbata* are believed to inhibit cancer cell proliferation and promote apoptosis. Other herbs, such as *Ganoderma lucidum* (Reishi) and *Astragalus membranaceus*, enhance immune function, helping patients resist diseases and reducing the risk of cancer recurrence. The anticancer mechanisms of TCM include cytotoxicity, immunomodulation 20, tumor metastasis suppression 21, 22, and gut microbiota modulation 23. TCM formulas are complex, involving multiple biological pathways. MWBS is a clinical auxiliary drug used in traditional Chinese medicine (TCM) for cancer treatment; however, its molecular mechanisms remain unclear. Our study showed that MWBSE can suppress the proliferation of various human cancer cell types. The anti-proliferative effect of MWBSE likely arises from cell cycle disruption via the targeting of Aurora kinases and Rb, providing new insights into the potential anticancer mechanisms of the clinically used formula, Modified Wu Bao San. The effective MWBSE concentrations (100-300 μg/mL) of our results were based on cells treated with MWBSE for 24 hours. In traditional Chinese medicine, anticancer treatments are typically administered continuously rather than as a single dose. Therefore, therapeutic concentrations in the body can be achieved through daily administration. Whether such concentrations are physiologically achievable *in vivo* should be re-evaluated through subsequent human clinical trials.

Treatment with low concentrations of MWBSE appears to induce G2/M phase arrest, while higher concentrations (300 μg/mL) arrest cells in the G1 phase, accompanied by a decreased S phase cell population (Fig. 2). According to flow cytometry data, MWBSE-treated cells are arrested in the G2/M and G1 phases. Further analysis of cell morphology following MWBSE treatment revealed that most cells exhibited a binucleated phenotype, suggesting cytokinesis failure. Cytokinesis is the final step of cell division, and its failure leads to multinucleated cells 18. Cells that experience mitotic or cytokinesis failure often undergo cell death 24. Seven signalling pathways regulate cytokinesis, with the chromosomal passenger complex (CPC) being the primary regulatory complex 25. Aurora B kinase, a component of the CPC, is essential for recruiting the centralspindlin complex to the spindle midzone, a necessary process for cytokinesis 26. We found that MWBSE treatment reduced phosphorylation of Aurora B kinase at T232—a modification required for its activation. By analysing the phosphorylation of the Aurora B substrate, histone H3 at Ser10, we confirmed that MWBSE inhibits Aurora B activity.

Aurora B kinase plays a critical role in mitosis, specifically in chromosome alignment, spindle checkpoint function, and cytokinesis. Due to its essential role in cell division, Aurora B has become a focus in cancer research, as uncontrolled cell proliferation is a hallmark of cancer. The overexpression of Aurora B kinase has been observed in various cancers, including breast, colorectal, and liver cancers. Elevated levels often correlate with increased cell proliferation and a greater risk of chromosomal instability, which can promote tumorigenesis 27. Given its roles and frequent upregulation in cancer, Aurora B kinase is a promising target for anticancer drugs 28, 29. The ability of MWBSE to inhibit Aurora B kinase offers a potential application in cancer treatment.

In addition to inducing cytokinesis failure, MWBSE can also arrest cells in the G1 phase. The retinoblastoma protein (Rb) is a key regulator of cell cycle progression 16. Rb functions primarily by inhibiting the transcription factor E2F, which controls the expression of genes required for DNA replication. In its active, hypophosphorylated form, Rb binds to E2F, preventing it from promoting the transcription of genes necessary for the G1/S phase transition 30. Mutations in the RB1 gene, resulting in loss of functional Rb, are directly linked to retinoblastoma, a rare childhood eye tumor 31. However, Rb dysfunction is implicated in many other cancer types 32. On the basis of the function of Rb, we hypothesized that G1 phase arrest in MWBSE-treated cells may be due to Rb activation. Our results showed that MWBSE treatment significantly reduced Rb phosphorylation. In addition to direct mutations, cancer cells often indirectly disrupt Rb function via hyperactivation of cyclin D-CDK4/6 complexes, leading to hyperphosphorylation of Rb and its inactivation. The overexpression of cyclin D or CDK4/6 is observed in several cancers, making CDK4/6 inhibitors an attractive therapeutic option 33. In this study, high concentrations of MWBSE reduced Rb phosphorylation at the Ser795 and Ser807/811 sites, leading to G0/G1 phase arrest and a decrease in proliferation rate. These results suggest that high concentrations of MWBSE may restore Rb function. We cannot exclude the possibility that MWBSE indirectly restores Rb function by inhibiting upstream kinases, as cyclin B protein levels were also reduced after MWBSE treatment. Additionally, Rb regulates apoptosis by binding to pro-apoptotic Bax at the mitochondria 34. Dephosphorylation of Rb at S807/S811, among other sites, causes Rb to dissociate from Bax, leading to a significant increase in apoptosis 35. Bax p18 was increased following MWBSE treatment, which aligns with the restoration of Rb function.

Aurora kinases are essential for mitosis and cytokinesis. Dysfunctional Aurora kinases cause mitotic defects, activating the spindle assembly checkpoint (SAC) pathway and arresting cells in mitosis. When cells cannot sustain prolonged M phase arrest, they may undergo cell death during mitosis (mitotic catastrophe) or slip into the G1 phase and die 28, 36, 37. Inhibition of Aurora kinases can trigger mitotic catastrophe, and inhibition of Aurora B, which is involved in both SAC and cytokinesis, can lead to mitotic slippage and cytokinesis failure. These studies support our observation that MWBSE-treated cells display a binucleated phenotype and undergo apoptosis, as MWBSE inhibits Aurora kinases. As an inhibitor of Aurora kinases and an activator of Rb, MWBSE shows promise as a potential anticancer treatment.

## Conclusion

**Figure 7:**
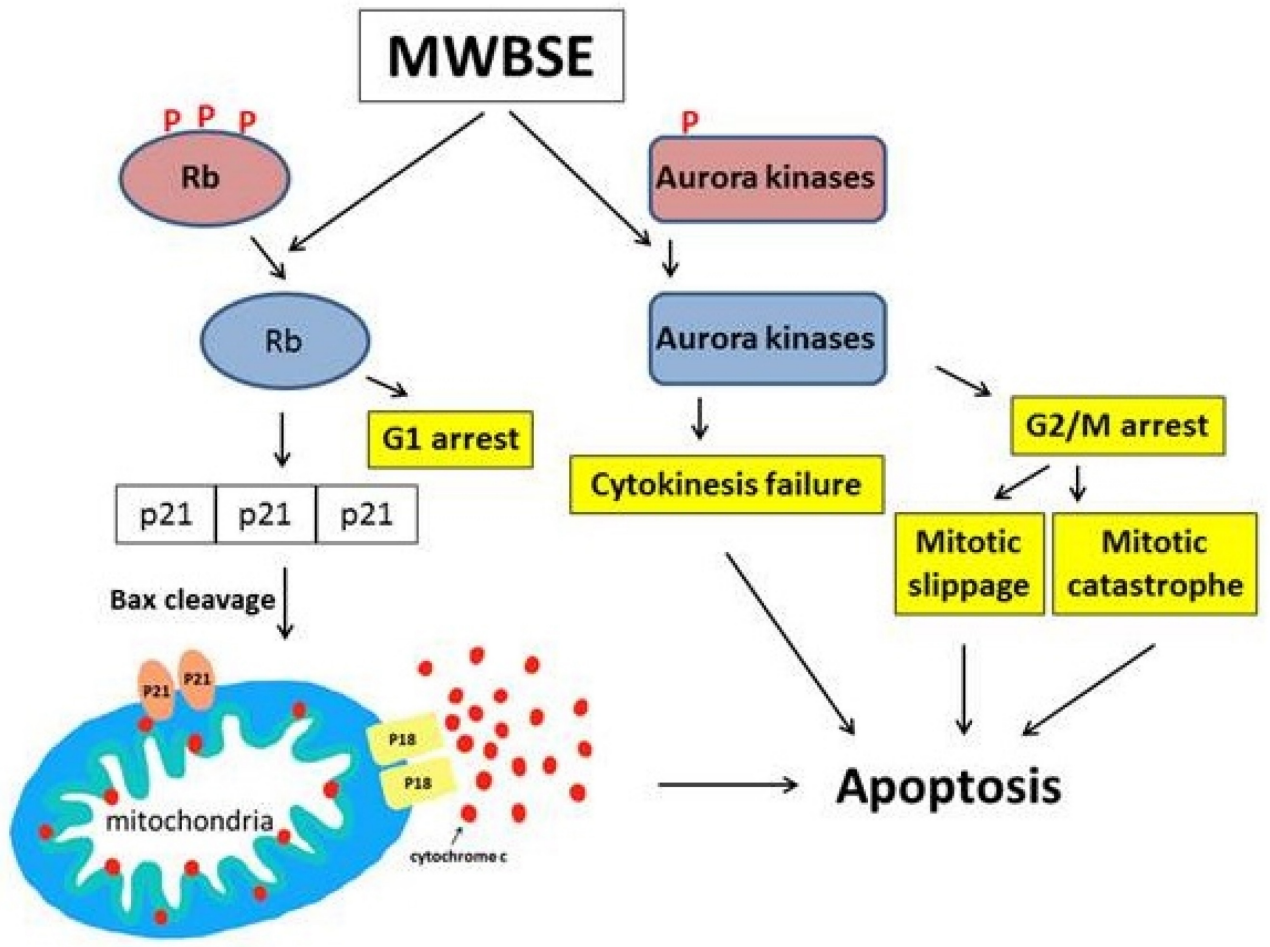
** Proposed model of the cell death pathways induced by MWBSE.** This schematic illustrates the proposed mechanisms through which MWBSE induces cell death, involving the inhibition of Aurora kinases, cytokinesis failure, cell cycle arrest, Rb dephosphorylation, and the activation of apoptosis pathways.

In this study, we demonstrated that MWBSE has multiple anticancer effects (Fig. 7). First, MWBSE induces cytokinesis failure by inhibiting Aurora kinase, leading cells to commit to cell death. Additionally, MWBSE reduces the phosphorylation of Rb, restoring its function and resulting in cell cycle arrest. Since both Aurora kinases and Rb are promising targets for cancer therapy, our findings suggest that MWBSE may serve as a potential agent in cancer treatment.
